# Stearoyl-CoA Desaturase-1 (SCD1) Augments Saturated Fatty Acid-Induced Lipid Accumulation and Inhibits Apoptosis in Cardiac Myocytes

**DOI:** 10.1371/journal.pone.0033283

**Published:** 2012-03-08

**Authors:** Hiroki Matsui, Tomoyuki Yokoyama, Kenichi Sekiguchi, Daisuke Iijima, Hiroaki Sunaga, Moeno Maniwa, Manabu Ueno, Tatsuya Iso, Masashi Arai, Masahiko Kurabayashi

**Affiliations:** 1 Department of Medicine and Biological Sciences, Gunma University Graduate School of Medicine, Showa-machi, Maebashi, Japan; 2 Department of Laboratory Sciences, Gunma University Graduate School of Health Sciences, Showa-machi, Maebashi, Japan; Pennington Biomedical Research Center, United States of America

## Abstract

Mismatch between the uptake and utilization of long-chain fatty acids in the myocardium leads to abnormally high intracellular fatty acid concentration, which ultimately induces myocardial dysfunction. Stearoyl-Coenzyme A desaturase-1 (SCD1) is a rate-limiting enzyme that converts saturated fatty acids (SFAs) to monounsaturated fatty acids. Previous studies have shown that SCD1-deficinent mice are protected from insulin resistance and diet-induced obesity; however, the role of SCD1 in the heart remains to be determined. We examined the expression of SCD1 in obese rat hearts induced by a sucrose-rich diet for 3 months. We also examined the effect of SCD1 on myocardial energy metabolism and apoptotic cell death in neonatal rat cardiac myocytes in the presence of SFAs. Here we showed that the expression of SCD1 increases 3.6-fold without measurable change in the expression of lipogenic genes in the heart of rats fed a high-sucrose diet. Forced SCD1 expression augmented palmitic acid-induced lipid accumulation, but attenuated excess fatty acid oxidation and restored reduced glucose oxidation. Of importance, SCD1 substantially inhibited SFA-induced caspase 3 activation, ceramide synthesis, diacylglycerol synthesis, apoptotic cell death, and mitochondrial reactive oxygen species (ROS) generation. Experiments using SCD1 siRNA confirmed these observations. Furthermore, we showed that exposure of cardiac myocytes to glucose and insulin induced SCD1 expression. Our results indicate that SCD1 is highly regulated by a metabolic syndrome component in the heart, and such induction of SCD1 serves to alleviate SFA-induced adverse fatty acid catabolism, and eventually to prevent SFAs-induced apoptosis.

## Introduction

Obesity and related metabolic diseases such as type 2 diabetes and metabolic syndrome are occurring in pandemic proportions. Obesity and diabetes have a propensity for increased circulating fatty acids that induce a distinct cardiac metabolic phenotype characterized by increased fatty acid uptake and β-oxidation [1]. Evidence from animal studies demonstrated that excessive fatty acid supply to the heart that is not accompanied by a parallel increase in fatty acid oxidation leads to lipotoxic cardiomyopathy characterized by the accumulation of triglycerides (TGs), ceramides and diacylglycerol (DAG), which are associated with increased myocyte apoptosis, myocardial fibrosis, and contractile dysfunction [1], [2]. In addition, cardiac steatosis, defined as excessive myocardial TG accumulation in the absence of left ventricular systolic dysfunction, has been demonstrated by ^1^H-magnetic resonance spectroscopy in type 2 diabetes patients [3].

Stearoyl-Coenzyme A desaturase-1 (SCD1) is a rate-limiting enzyme that converts saturated fatty acids (SFAs) into monounsaturated fatty acids (MUFAs), mainly oleate (18∶1) and palmitoleate (16∶1). These represent the major MUFAs of membrane phospholipids, TGs, and cholesterol esters (CEs) [4]. Previous studies have shown that SCD1-deficinent mice are lean and are protected from insulin resistance, hypertriglyceridemia, hepatic steatosis, and diet-induced and genetically induced obesity [5]. The reduction of plasma TG in SCD1-deficient mice is mediated largely through increased fatty acid oxidation and reduced fatty acid synthesis in the liver [6], [7]. Furthermore, it has been well described that SFAs are potent proinflammatory molecules and increase the expression of a number of inflammatory genes by a nuclear factor kB (NFkB)-dependent mechanism in adipocytes and macrophages via Toll-like receptor 4 (TLR4) [8], [9], [10]. Moreover, systemic administration of antisense oligonucleotide directed against SCD1 mRNA efficiently prevents the onset of hepatic insulin resistance in high-fat-fed rats [11]. These studies imply that SCD1 is a critical mediator of metabolic syndrome; however, unexpectedly, the absence of SCD1 either by gene deletion or antisense oligonucleotide accelerated atherosclerosis in a mouse model of hyperlipidemia and atherosclerosis on a Western diet despite relatively reduced plasma TG and increased insulin sensitivity [12], [13]. These adverse effects are likely derived from increased atherogenic inflammation of the arterial wall due to increased circulating VLDL, which are highly enriched SFAs. These results suggest that intracellular levels of SFAs and MUFAs controlled by SCD1 have a distinct impact on cellular function in a cell-type-dependent manner and SCD1 expression should be tightly controlled.

In this study, we aimed to determine the role of SCD1 and inducible stimuli of SCD1 gene expression in the heart. We employed visceral obese rats fed a high-sucrose (HS) diet as a model of metabolic syndrome and found a marked increase in SCD1 expression in the heart. In addition, using adenovirus and siRNA to alter SCD1 expression in cultured cardiac myocytes, we found evidence indicating that the induction of SCD1 in the heart markedly inhibits SFA-induced caspase 3 activation, ceremide synthesis, DAG synthesis, apoptosis and mitochondrial reactive oxygen species (ROS) generation, and thus seems to act to prevent SFA-induced lipotoxic cardiomyopathy.

## Results

### HS diet induced visceral obesity, but did not affect cardiac function

As shown in Table 1, the HS diet group was 10% heavier than the chow-fed group (p<0.05). Total visceral fat weight was 94% higher in the HS diet group than in the chow group after 3 months of feeding (Table 1); however, there were no differences in heart weight or carotid systolic and diastolic pressures between the two groups. Furthermore, echocardiographic parameters, such as left ventricular wall thickness and left ventricular functional parameters (EF, FS, E/A ratio) were not statistically different between two groups (Table 1). These data indicate that the 3-month HS diet induced severe visceral obesity but abnormalities of the cardiac structure and function were not apparent.

**Table 1:** Biometric and echocardiographic parameters of rats fed the control or HS diet.

	Control	High sucrose
Physical measurements		
Body weight (g)	438.5±47.5	486.0±31.7*
Heart weight (g)	0.88±0.08	0.94±0.06
SBP (mmHg)	100.1±20.8	99.0±11.7
DBP (mmHg)	76.1±24.6	66.6±15.5
Visceral Fat (g)	15.9±3.2	31.0±3.9**
Echocardiogram		
IVSd (mm)	1.7±0.3	1.8±0.3
LVEdD (mm)	8.3±0.6	7.7±0.9
LVPWd (mm)	1.7±0.2	1.9±0.3
LVEsD (mm)	5.1±0.6	4.5±1.1
EF (%)	74.1±6.9	76.9±8.4
FS (%)	38.9±5.5	41.8±9.1
E/A	2.0±0.2	2.1±0.6

Data are the means ± SD.

* P<0.05, **P<0.01 vs. control fed group.

SBP, systolic blood pressure; DBP, diastolic blood pressure; IVSd, Interventricular septal end-diastolic dimension; LVEdD or LVEsD, left ventricular end-diastolic or systolic diameter; LVPWd, left ventricular posterior wall thickness in diastole; EF, ejection fraction; FS, fractional shortening; E/A, transmitral flow ratio.

### HS diet induced SCD1 mRNA expressions in rat hearts

**Figure 1:**
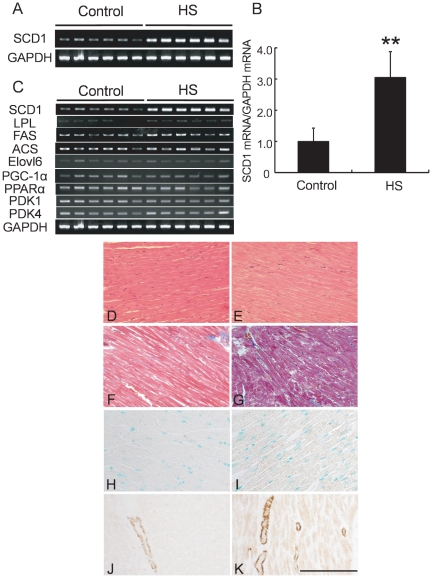
Expression of SCD1 in HS diet rat hearts and in obese and diabetes patients' hearts. (A) and (B): Semi-quantitative RT-PCR (A) and real-time RT-PCR (B) showed the increased expression of SCD1 after 3 months in HS diet rat hearts in comparison with normal fed rat hearts. GAPDH was measured as an internal control. mRNA of the normal fed group was normalized to a value of 1, and the mRNA level in the HS-fed group is shown relative to the control level. Values are reported as the means ± SD. N = 6–8, **p<0.01 vs. control. (C): Semi-quantitative RT-PCR of SCD1, lipoprotein lipase (LPL), fatty acid synthase (FAS), acyl-CoA synthase (ACS), elongation of long chain fatty acid member 6 (Elovl6), peroxisome proliferator coactivator-1α (PGC-1α), peroxisome proliferator-activated receptor α (PPARα), pyruvate dehydrogenase kinase isozyme 1 and 4 (PDK1, PDK4) in HS- or normal fed rat hearts after 3 months. Expression levels of other lipid biosynthesis genes or FA oxidative genes were similar between two groups at the same time point. (D) to (G): Pathology of SCD1 in HS- or normal fed rat heart. HE-stained sections (D) and (E) and Masson's trichrome staining (F) and (G) of rat myocardium after 3 months of feeding. (D), (F): Normal fed; (E), (G): High-sucrose fed. (H) and (I): Immunohistochemical staining showed increased expression of SCD1 after 3 months in HS-fed rat hearts (I), while minimal SCD1 staining was observed in control hearts (H). (J) and (K): Although SCD1 was not detectable in cardiac myocytes of normal healthy subject (J) using immunohistochemical staining, SCD1 expression was increased in obese and diabetes patients' hearts (K). Counterstaining was performed with 2% methyl green. Scale bar = 200 µm.

To determine the effects of HS diet on the expression of SCD1 in rat hearts, SCD1 mRNA expression was determined by semi-quantitative or real-time RT-PCR after 3 months of feeding. As shown in Figure 1A and B, SCD1 mRNA was significantly increased after HS feeding (2.0±0.4 fold. vs control. n = 6, *p<0.05). In contrast, expression levels of other lipid biosynthesis genes or FA oxidative genes were similar between control and HS diet groups (Figure 1C) at the same time point. Therefore, SCD1 was specifically induced in HS-fed rat hearts.

### HS diet or obesity induced SCD1 protein expression in rat and human hearts

Analysis of histological change with hematoxylin and eosin (HE) or Masson's trichrome staining did not reveal differences in morphology between control and HS diet groups (Figure 1D–G). To determine the cellular location of SCD1 proteins in rat hearts, we performed immunohistochemical staining of ventricular sections with anti-SCD1 antibodies after 3 months of feeding. While minimal SCD1 staining was observed in the control hearts (Figure 1H), SCD1 was clearly stained in the cytoplasm of HS diet rat hearts after 3 months of feeding (Figure 1I). Furthermore, we examined the human hearts obtained by autopsy. The expression of SCD1 appears to be more abundant in the hearts of diabetic patients than in those from lean subjects and non-diabetics (Figure 1J and K). These observations suggest that the myocardial expression of SCD1 increases in obesity.

### HS diet increased plasma glucose, insulin and FFAs levels, and plasma/tissue SCD1 activity

Plasma glucose levels, insulin levels and HOMA-IR were higher in rats fed the HS diet than rats fed the normal diet (Table 2). Plasma FFA levels also increased in the HS diet group. In contrast, other lipid parameters, such as HDL cholesterol, LDL cholesterol, total cholesterol and TG, were comparable between the two groups. Furthermore, analysis of plasma and heart tissue FFA composition showed that the percentage of MUFAs, such as palmitoleic acid or oleic acid, was markedly increased, while that of stearic acid, an SFA, was decreased (% by weight, Table 3, 4). From these FFA composition analyses, the ratio of oleic acid (18∶1 ω9) to stearic acid (18∶0) was also significantly increased in rat hearts with HS diet compared to control animals (Table 3,4). These results proved that HS diet increased SCD1 activity in rat hearts as well as plasma SCD1 activity. In addition, TG content in the hearts was significantly increased after HS feeding in comparison with control rat hearts (1.70±0.05 vs. 1.37±0.05 mg/g tissue, n = 4, p<0.05). These results are consistent with the increased expression of SCD1 in rats fed the HS diet.

**Table 2:** Plasma parameters of rats fed the control or HS diet for 3 months.

	Control	High sucrose
HDL (mg/dl)	24.6±5.4	24.4±3.1
LDL (mg/dl)	4.4±1.8	3.7±0.8
Total cholesterol (mM/l)	1.4±0.4	1.3±0.2
Triglyceride (mg/dl)	20.7±21.6	50.7±61.0
Free fatty acid (μEq/l)	576.4±344.2	2241.8±869.3**
Glucose (mg/dl)	167.2±33.6	176.7±22.2**
Insulin (ng/ml)	3.6±2.5	11.3±4.9**

Data are the means ±SD. **P<0.01 vs. control fed group.

** HDL: high density lipoprotein, LDL: low density lipoprotein.

**Table 3:** Plasma fatty acid composition of rats fed the control or HS diet for 3 months.

	Control	High-sucrose
Lauric acid (C12∶0)	0.0±0.0	0.0±0.0
Myristic acid (C14∶0)	0.3±0.1	0.6±0.1*
Myristoleic acid (C14∶1 ω5)	<0.01	<0.01
Palmitic acid (C16∶0)	23.2±1.3	24.6±0.8
Palitoleic acid (C16∶1 ω7)	2.0±0.2	6.9±2.3*
Stearic acid (C18∶0)	10.9±0.2	7.9±1.6**
Oleic acid (C18∶1 ω9)	9.6±0.7	18.7±2.9**
Linoleic acid (C18∶2 ω6)	17.5±1.3	13.6±1.4**
γ-linolenic acid (C18∶3 ω6)	0.4±0.1	0.3±0.0
Linolenic acid (C18∶3 ω3)	0.3±0.1	0.3±0.0
Arachic acid (C20∶0)	0.1±0.0	0.1±0.0
Eicosenoic acid (C20∶1 ω9)	0.1±0.0	0.2±0.0*
Eicosadienoic acid (C20∶2 ω6)	0.1±0.0	0.2±0.0*
5-8-11eicosatrienoic acid (C20∶3 ω9)	0.1±0.0	0.5±0.1*
Dihomo-γ-linolenic acid (C20∶3 ω6)	0.6±0.1	1.2±0.2**
Arachidonic acid (C20∶4 ω6)	27.5±2.5	17.4±5.9*
Eicosapentaenoic acid (C20∶5 ω3)	0.6±0.1	1.0±0.4
Behenic acid (C22∶0)	0.3±0.0	0.3±0.0*
Erucic acid (C22∶1 ω9)	<0.01	<0.01
Docosatetraenoic acid (C22∶4 ω6)	0.3±0.0	0.2±0.0
Docosapentaenoic acid (C22∶5 ω3)	0.6±0.1	0.6±0.2
Lignoceric acid (C24∶0)	0.8±0.1	0.6±0.1*
Docosaphexaenoic acid (C22∶6 ω3)	4.2±0.4	4.3±0.2
Nervonic acid (C24∶1 ω9)	0.6±0.1	0.6±0.1
SCD1 activity (18∶1 ω9/18∶0)	0.9±0.1	2.5±0.8**

Data are the means ±SD, and are presented as percent of total fatty acids.

* P<0.05, **P<0.01 vs. control fed group.

**Table 4:** Heart tissue fatty acid composition of rats fed the control or HS diet for 3 months.

	Control	High-sucrose
Lauric acid (C12∶0)	0.0±0.0	0.0±0.0
Myristic acid (C14∶0)	0.1±0.0	0.3±0.1*
Myristoleic acid (C14∶1 ω5)	<0.01	<0.01
Palmitic acid (C16∶0)	13.2±1.1	14.8±1.0
Palitoleic acid (C16∶1 ω7)	0.4±0.1	1.7±0.5**
Stearic acid (C18∶0)	20.2±0.8	18.0±1.1*
Oleic acid (C18∶1 ω9)	4.8±0.7	7.6±1.4**
Linoleic acid (C18∶2 ω6)	24.8±1.2	24.2±2.1
γ-linolenic acid (C18∶3 ω6)	0.2±0.0	0.1±0.0*
Linolenic acid (C18∶3 ω3)	0.2±0.0	0.1±0.0
Arachic acid (C20∶0)	0.3±0.1	0.3±0.0
Eicosenoic acid (C20∶1 ω9)	0.1±0.0	0.1±0.0
Eicosadienoic acid (C20∶2 ω6)	0.2±0.0	0.1±0.0*
5-8-11eicosatrienoic acid (C20∶3 ω9)	<0.01	0.1±0.0
Dihomo-γ-linolenic acid (C20∶3 ω6)	0.4±0.0	0.6±0.1**
Arachidonic acid (C20∶4 ω6)	19.3±1.1	17.9±1.2
Eicosapentaenoic acid (C20∶5 ω3)	0.2±0.0	0.2±0.0
Behenic acid (C22∶0)	0.3±0.1	0.2±0.0
Erucic acid (C22∶1 ω9)	<0.01	<0.01
Docosatetraenoic acid (C22∶4 ω6)	0.7±0.1	0.6±0.0
Docosapentaenoic acid (C22∶5 ω3)	2.0±0.3	1.8±0.2
Lignoceric acid (C24∶0)	0.3±0.0	0.3±0.0
Docosaphexaenoic acid (C22∶6 ω3)	12.2±1.2	10.7±1.3
Nervonic acid (C24∶1 ω9)	0.1±0.0	0.1±0.0
SCD1 activity (18∶1 ω9/18∶0)	0.2±0.0	0.4±0.1*

Data are the means ±SD, and are presented as percent of total fatty acids.

* P<0.05, **P<0.01 vs. control fed group.

### Glucose, insulin, and saturated FAs induced SCD1 expression in cardiac myocytes

**Figure 2:**
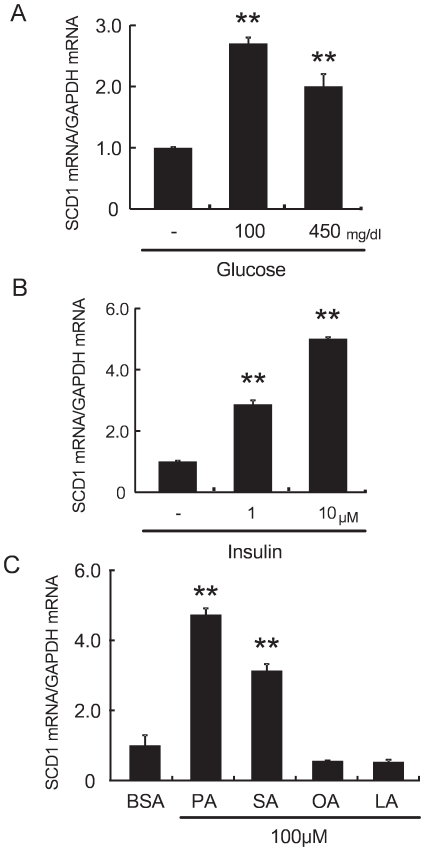
Effect of glucose, insulin and FFAs on SCD1 mRNA expression in neonatal rat cardiac myocytes. (A) to (C): Real-time RT-PCR showed that SCD1 expressions were increased in neonatal rat cardiac myocytes which treated with (A) glucose (100 to 450 mg/dl), (B) insulin (1 to 10 µM), and (C) saturated FAs, palmitic acid (PA) or stearic acid (SA) for 24 h. On the other hand, unsaturated FAs, oleic acid (OA) or linoleic acid (LA) didn't affect the expression of SCD1.

To determine the pathogenic factors contributing to the increase in SCD1 expression in the heart, neonatal rat cardiac myocytes were incubated with glucose, insulin, SFAs or MUFAs. SCD1 mRNA expression was increased by glucose or insulin in a dose-dependent manner (Figure 2A and B). Furthermore, SFAs, palmitic acid or stearic acid induced SCD1 mRNA expression. In contrast, MUFAs, oleic acid or linoleic acid didn't affect the expression of SCD1 (Figure 2C). These results are consistent with our in vivo data indicating that an induction of SCD1 mRNA in the heart was associated with an increase in plasma glucose, insulin and FFAs levels in obese rats.

### SCD1-overexpression in cardiac myocytes induced lipid accumulation, and suppressed saturated FA-induced excessive FA oxidation

**Figure 3:**
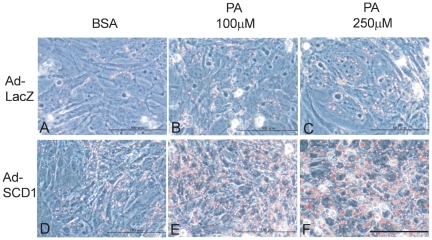
Involvement of SCD1 and lipid accumulation in cardiac myocyte. (A) to (F): Neonatal rat cardiac myocytes were infected with Ad-LacZ or Ad-SCD1 at a multiplicity of infection of 20. Oil red O staining showed that TG accumulation was markedly increased in myocytes transduced with Ad-SCD1 (D to F) in comparison with cells transduced with Ad-LacZ (A to C). 100 µM (B and E) or 250 µM (C and F) addition of palmitic acid (PA) was used as a substrate of SCD1, and BSA was used as a vehicle. Scale bar = 100 µm.

**Figure 4:**
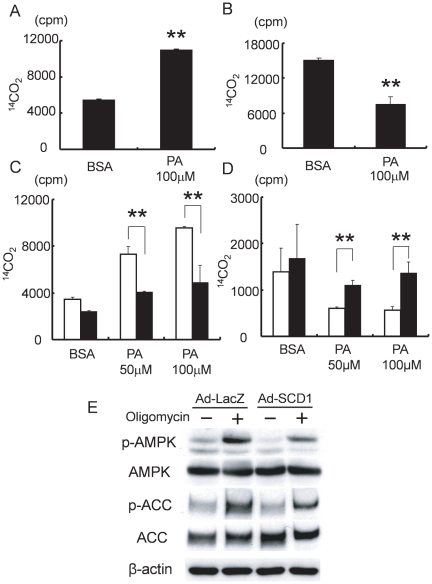
FA and glucose oxidations in SCD1-overexpressed cardiac myocytes. (A): FA oxidation was significantly increased in rat neonatal cardiac myocytes treated with 100 µM palmitic acid (PA). (B): On the other hand, glucose oxidation was significantly reduced in cardiac myocytes treated with PA (100 µM). (C): PA (50 to 100 µM) induced excessive FA oxidation was attenuated in cardiac myocytes transduced with a MOI of 20 of Ad-SCD1 (▪) in comparison with control cells transduced with Ad-LacZ (□). (D): PA (50 to 100 µM) -induced suppression of glucose oxidation recovered in cardiac myocytes transduced with Ad-SCD1 (▪) in comparison with control cells transduced with Ad-LacZ (□). BSA was used as a vehicle. Each sample was counted in a scintillation counter (cpm) and data are shown as the mean ± SD. **P<0.01 vs. Ad-LacZ in each PA concentration. (E); Phosphorylation levels of AMPK and ACC decreased in cardiac myocytes transduced with an MOI of 20 of Ad-SCD1 in comparison with control cells transduced with Ad-LacZ. Oligomycin (1 µM) was used as an AMPK and ACC activator.

To determine the effects of SCD1 on fatty acid homeostasis in cardiac myocytes, we prepared SCD1-overexpressing cardiac myocytes using adenovirus. Palmitic acid was used as a substrate of SCD1. As shown in Figure 3, adenovirus-mediated SCD1 overexpression markedly induced lipid accumulation by the addition of palmitic acid compared with Ad-LacZ-transduced cells. We next examined the effects of SCD1 on the oxidation of FA and glucose in cardiac myocytes. Consistent with the previous report, palmitic acid markedly increased FA oxidation and decreased glucose oxidation in the absence of SCD1 overexpression (Figure 4A and B). Interestingly, as shown in Figure 4C and D, the overexpression of SCD1 significantly attenuated palmitic acid-induced FA oxidation, and restored palmitic acid-induced suppression of glucose oxidation. These results suggest that elevated SCD1 expression ameliorates SFA-induced excessive energy reliance on FA.

Because β-FA oxidation is stimulated by AMP-activated protein kinase (AMPK), we examined whether the inhibition of palmitic acid-induced FA β-oxidation in SCD1 overexpression is mediated by the suppression of AMPK activity. In addition, we examined the phosphorylation of acetyl-CoA carboxylase beta (ACCβ) at Ser-79, which leads to inactivation of ACC activity and decreases malonyl-CoA synthesis. Because malonyl-CoA strongly inhibits the activity of CPT-I, which plays a key role in controlling the rate of FA uptake by mitochondria, AMPK activation and subsequent ACCβ-phosphorylation result in the acceleration of fatty acid oxidation. The results showed that SCD1 overexpression decreased the phosphorylation of AMPK and ACCβ in the absence or presence of an AMPK-activating agent, oligomycin (Figure 4E). Therefore, we assumed that attenuation of SFA-induced up-regulation of FA oxidation is mediated at least partly through the inhibition of AMPK activity and the resultant increased ACC activity.

### SCD1 attenuated saturated FA-induced apoptosis in cardiac myocytes

**Figure 5:**
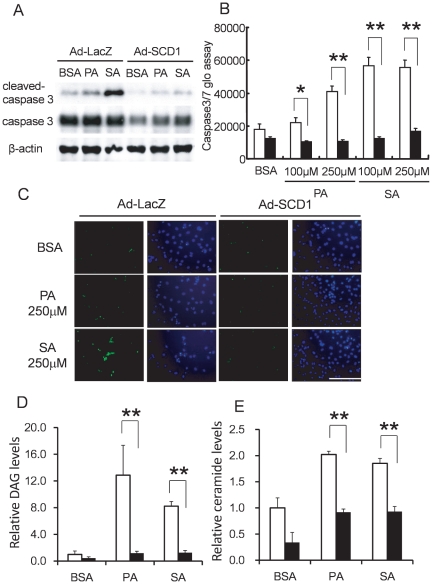
Saturated FA-induced apoptosis, DAG and ceramide level in SCD1-overexpressed cardiac myocytes. Cardiac myocytes were infected with Ad-LacZ as a control or Ad-SCD1 at an MOI of 20, and simultaneously treated with palmitic acid (PA) or stearic acid (SA). (A): Cleaved-caspase 3 and caspase 3 protein levels were analyzed by Western blotting. 250 µM of PA or SA induced cleaved-caspase 3 levels, and these inductions were attenuated in myocytes transduced with Ad-SCD1. (B): Caspase 3 and 7 activities were increased with PA or SA (100 to 250 µM) in cardiac myocytes, and inhibited in cells transduced with Ad-SCD1 (▪) in comparison with control cells transduced with Ad-LacZ(□). Values are shown as the mean ± SD. **P<0.01 vs. Ad-LacZ in each PA or SA concentration. (C): To detect cell death via DNA fragmentation, left panels illustrate TUNEL fluorescent staining (green) and right panel illustrates nuclei (DAPI: blue). Scale bar = 200 µm. 250 µM of PA or SA induced TUNEL-positive cells in neonatal rat cardiac myocytes transduced with Ad-LacZ, and these TUNEL-positive cells were reduced by Ad-SCD1 transduction. (D) and (E): Both (D) DAG and (E) ceramide levels in lipid extracts were significantly increased by the addition of PA or SA (250 µM), and these increases of DAG and ceramide were attenuated in myocytes transduced with Ad-SCD1 (▪) in comparison with control cells transduced with Ad-LacZ (□). Each sample was counted in a scintillation counter (cpm). An arbitrary value of 1.0 was assigned to cells transduced with Ad-LacZ by the addition of BSA. Data are shown as the mean ± SD. **P<0.01 vs. Ad-LacZ in each PA or SA concentration.

We next examined whether SCD1 regulates saturated FA-induced apoptosis. As shown in Figure 5A–C, palmitic acid or stearic acid increased the activated caspase 3 protein level, caspase 3 and 7 activities, and TUNEL-positive stained cells. In contrast, SCD1 overexpression significantly attenuated saturated FA-induced apoptotic changes. To determine the mechanisms by which SCD1 mediates the modification of saturated FA-induced apoptosis, we assayed DAG and ceramide levels in lipid extracts of cardiac myocytes transduced with Ad-LacZ or Ad-SCD1 by the addition of saturated FAs. Both DAG and ceramide levels were markedly induced by stimulation with palmitic acid or stearic acid, while these effects were blunted by SCD1 overexpression using Ad-SCD1 (Figure 5D and E). These results suggest that SCD1 suppressed saturated FA-induced DAG and ceramide production in cardiac myocytes.

**Figure 6:**
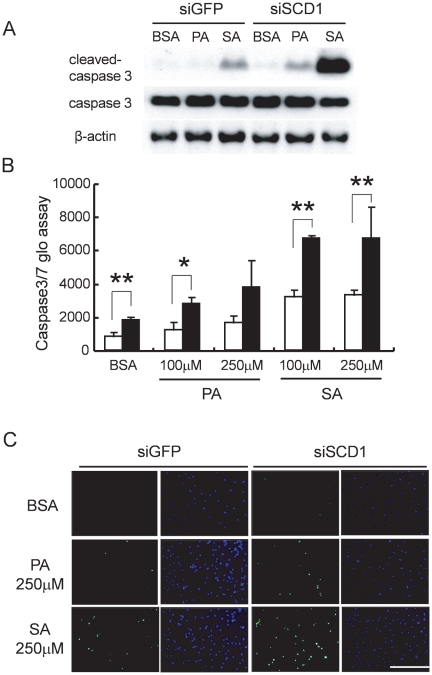
Saturated FA-induced apoptosis in cardiac myocytes with loss of SCD1 function using siRNA transfection. Cardiac myocytes were transfected with siGFP as a control or siSCD1 at 20 µM, and simultaneously treated with palmitic acid (PA) or stearic acid (SA). (A): Loss of SCD1 function (siSCD1) augmented cleaved-caspase 3 levels induced by 250 µM of PA or SA. (B): Caspase 3 and 7 activities induced by PA or SA (100 to 250 µM) in cardiac myocytes were also augmented by the loss of SCD1 function (siSCD1: ▪) in comparison with control cells transduced with siGFP (□). Values are shown as the mean ± SD. *P<0.05 or **P<0.01 vs. siGFP in each PA or SA concentration. (C): 250 µM of PA or SA induced TUNEL-positive cells in neonatal rat cardiac myocytes transduced with siGFP, and these TUNEL-positive cells increased by the loss of SCD1 function (siSCD1). Scale bar = 200 µm.

To confirm the suppressive effects of SCD1 on saturated FA-induced apoptosis, we studied the effects of loss of function of SCD1 using siRNA transfection in cardiac myocytes. As expected, FA induced-apoptotic changes, such as the caspase 3 level, caspase activity, and TUNEL-positive cells, were augmented by knockdown of SCD1 (Figure 6A–C). These findings suggest that SCD1 had protective effects against saturated FA-induced apoptosis in cardiac myocytes.

### SCD1 modulated saturated FA-induced ROS levels in cardiac myocytes

**Figure 7:**
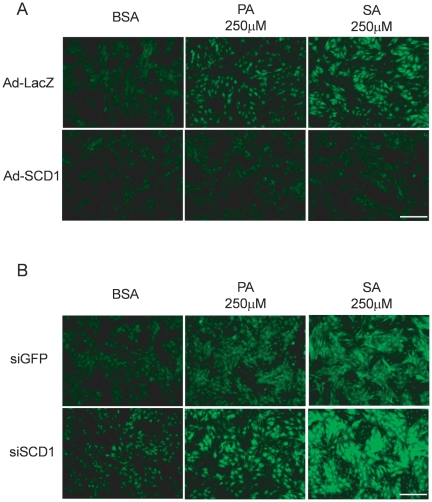
SCD1 modulated saturated FA-induced ROS levels in cardiac myocytes. Cardiac myocytes were (A) infected with Ad-LacZ as a control or Ad-SCD1 at an MOI of 20 or (B) transfected with siGFP as a control or siSCD1 at 20 µM, and simultaneously treated with palmitic acid (PA, 250 µM) or stearic acid (SA, 250 µM). Intracellular ROS levels were detected with CM-H_2_DCFDA. PA or SA-induced ROS levels in neonatal rat cardiac myocytes transduced with Ad-LacZ, and these ROS levels were reduced by Ad-SCD1 transduction. In contrast, knockdown of SCD1 by siRNA increased basal ROS levels, and enhanced PA or SA-induced ROS levels. Scale bar = 200 µm.

To assess the protective effects of SCD1 on mitochondrial function from saturated FA toxicity in cardiac myocytes, we tested mitochondrial ROS levels induced by PA or SA in cardiac myocytes. Cardiac mycytes were infected with Ad-LacZ or Ad-SCD1, transfected with siGFP or siSCD1 in the presence or absence of PA or SA; ROS levels were detected in the treated cells. As shown in Figure 7A, Ad-LacZ infection alone had minimal effects on basal ROS levels. PA and SA significantly increased intracellular ROS levels in cardiac myocytes transduced with Ad-LacZ, an observation consistent with previous reports [14]. These ROS levels were markedly reduced by adenovirus-mediated SCD1 overexpression. In contrast, suppression of SCD1 by specific siRNA not only increased basal ROS levels, but also augmented the PA or SA–induced increase in ROS levels (Figure 7B). These data suggest that SCD1 expression was closely related to protection of mitochondria from PA and SA-induced toxicity.

### High dose of hydrogen peroxide decreased SCD1 mRNA expression in cardiac myocytes

**Figure 8:**
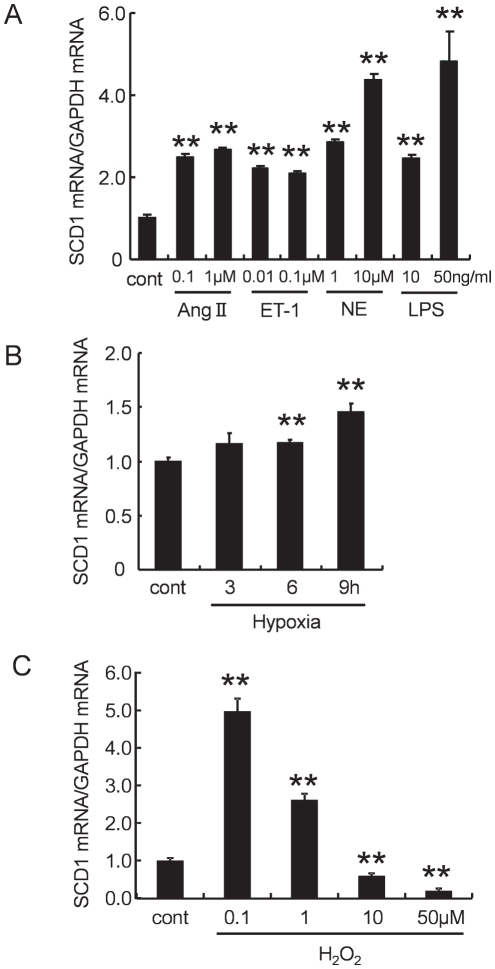
Effect of several humoral factors on SCD1 mRNA expression in neonatal rat cardiac myocytes. Real-time RT-PCR of SCD1 expressions in neonatal rat cardiac myocytes stimulated with (A) angiotensin II (0.1 to 1 µM), endothelin-1 (0.01 to 0.1 µM), norepinephrine (1 to 10 µM), lipopolysaccharide (10 to 50 ng/ml) for 24 h, (B) hypoxia (3 to 9 h) and (C) Hydrogen peroxide (H_2_O_2_: 0.1 to 50 µM) for 24 h. High dose (10–50 µM) of hydrogen peroxide significantly inhibited SCD1 mRNA expression in neonatal rat cardiac myocytes. Each SCD1 expression is presented relative to the gene expression of GAPDH and control is normalized to a value of 1. Values are reported as the means ± SD. **p<0.01 vs. control.

These results of *in vivo* and *in vitro* experiments led us to propose a new model in which SCD1 exerts a protective effect against saturated FAs-induced cardiac lipotoxicity. We next tried to identify factors that regulate SCD1 gene expressions in cardiac myocytes. Isolated cardiac myocytes were incubated with various reagents which are relevant to oxidative stress, such as angiotensin II, endothelin-1, norepinephrine, lipopolysaccharide, hypoxia, and hydrogen peroxide. Real-time PCR revealed that all of the reagents tested significantly increased SCD1 mRNA expression (Figure 8A and B). Low dose (0.1–1 µM) of hydrogen peroxide, an analog of oxidative stress, also significantly increased SCD1 mRNA expression in cardiac myocytes, however, high dose (10–50 µM) of hydrogen peroxide markedly decreased SCD1 mRNA expression (Figure 8C). Thus, the strong oxidative stress induced by such as a high dose of hydrogen peroxide may inhibit SCD1 expression in cardiac myocytes.

## Discussion

The major findings in this study are five-fold. First, we provide evidence that SCD1 expression and activity are markedly induced in the heart of obese diabetic rats fed an HS diet. Second, by in vitro experiments using an adenovirus- or siRNA-mediated approach, we show that SCD1 plays a role in attenuating palmitic acid-induced excessive fatty acid oxidation and caspase 3 activation that leads to apoptosis in neonatal rat cardiac myocytes. Third, SCD1 overexpression inhibits palmitic acid-induced de novo synthesis of ceramide and DAG. Fourth, SCD1 attenuates palmitic acid-induced mitochondrial ROS generation in cardiac myocytes. Fifth, SCD1 expression in cardiac myocytes is highly sensitive to a number of dietary, hormonal, and environmental factors. Interestingly, hydrogen peroxide, an inducer of oxidative stress, has the positive or negative effect on SCD1 expression depending on its concentration similar to a previous report [15]. Collectively, these findings suggest that the induction of SCD1 expression in the heart serves to protect the cells from the unfavorable effects of elevated SFAs, which cause lipotoxic cardiomyopathy.

### SCD1 is a highly regulated gene in the heart

We found that SCD1 mRNA levels most markedly increased in the heart by the HS diet. Among the many genes involved in lipogenesis, the SCD1 gene seems to be specifically activated in the heart because the expression of other genes examined, such as LPL, FAS, and ACS genes, was not measurably affected. Notably, the expression of PPARα, PGC-1α and PGC-1β, inducible nuclear receptor and its coactivators, which regulate the genes encoding enzymes at every level of oxidative energy metabolism including fatty acid uptake and mitochondrial β-oxidation [16], [17], was not induced by the HS diet. Although we did not address the rationale for this differential regulation among lipogenic genes by the HS diet, we assume that in the heart, HS diet preferentially enhances the pathway that leads to fatty acid storage as TG or CE rather than fatty acid synthesis, given that SCD1 catalyzes the biosynthesis of MUFAs from SFAs, and MUFAs serve as the preferred substrate for the synthesis of storage lipids, TG and CE [18].

### Is SCD1 in the heart a friend or foe?

**Figure 9:**
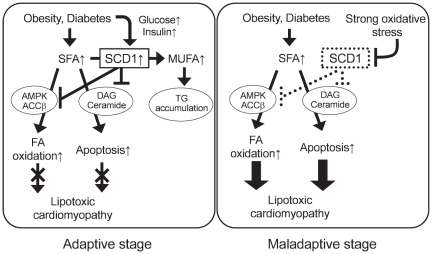
A proposed model for the protective role of SCD1 against FA-induced cardiac lipotoxicity. *Adaptive stage*: SFAs uptake increases in the myocardium in obesity and diabetes, and SCD1 expression is induced to convert SFAs to MUFAs. Increased SCD1 expression in turn inhibits FA oxidation and apoptosis through an inhibition of AMPK/ACCβ activity and DAG/ceramide synthesis, which protect the heart from lipotoxic cardiomyopathy. *Maladaptive stage*: excessive humoral and nutrient stimuli increase oxidative stress which reduces SCD1 expression. As a consequence, pathways leading to FA oxidation and apoptosis are facilitated and lipotoxic cardiomyopathy occurs.

Our findings that SCD1 overexpression reduced SFA-induced apoptosis, and that siRNA for SCD1 abolished this protective effect allowed us to hypothesize that SCD1 induction is beneficial rather than detrimental to the heart. As illustrated in Figure 9, elevated plasma FA, glucose and insulin increased SCD1 expression in cardiac myocytes. Increased expression of SCD1, in turn, might protect from palmitic acid-induced excessive fatty acid oxidation and apoptosis through mechanisms involving down-regulation of the AMPK/ACC-pathway, inhibition of ceramide and DAG synthesis, and inhibition of mitochondrial ROS generation. Furthermore, mild oxidative stress may stimulate SCD1 expression. We suggest that this period is an adaptive stage. On the other hand, SCD1 expression is reduced when oxidative stress is increased. In this condition, SFAs are no longer converted to MUFAs, and ceramide synthesis increases and, as a consequence, strong oxidative stress contributes to the onset of lipotoxic cardiomyopathy. Thus, we propose this as a maladaptive stage.

This concept is clearly contradictory to previous reports, which showed that SCD1 deficiency in the liver and adipose tissue [19] as well as in the whole body results in a marked beneficial metabolic outcome, including increased energy expenditure, reduced body adiposity and increased insulin sensitivity [6], [7]. In addition, Hulver et al. demonstrated that SCD1 gene expression is up-regulated in skeletal muscle from extremely obese humans [20]. They showed that elevated SCD1 expression increased TG synthesis and MUFAs in skeletal muscle because overexpression of SCD1 in cultured primary skeletal muscle cells from lean subjects produced a metabolic phenotype resembling that displayed by myocytes from obese subjects; increased TG synthesis and decreased fatty acid oxidation. Inhibition of SCD activity has been proposed as a therapeutic strategy for the treatment of obesity, diabetes, liver steatosis and metabolic syndrome [21]; however, the ability of SCD1 to prevent SFA-induced apoptosis of cardiac myocytes suggests that SCD1 in the heart is a potential target that should be activated, at least in the short term, for the prevention of apoptosis.

### Effects of SCD1 on palmitic acid-induced fatty acid oxidation and glucose oxidation in the heart

We envisage several mechanisms through which SCD1 overexpression attenuates palmitic acid-induced fatty oxidation. First, SCD1 channels palmitic acid toward the storage of TG and away from β-oxidation, as demonstrated by previous studies using SCD1-overexpressing CHO cells and primary skeletal muscle [20], [22], [23]. In those studies, MUFAs but not SFAs are readily incorporated into TG while SFAs do not lead to TG accumulation. Second, we found that SCD1 overexpression decreased the phosphorylation of AMPK. Consistent with the results of this study, inhibition of AMPK phosphorylation by SCD1 has been described [20]. In that study, SCD1 overexpression in primary human skeletal muscle cells decreased fatty acid oxidation by 45% and reduced AMPK and ACC activity. Furthermore, SCD1 deficiency activated AMPK in the liver and skeletal muscle but not in the heart [24], [25], [26]. Further studies are necessary to determine the mechanisms by which SCD1 controls AMPK activity in the heart.

We found that exogenous palmitic acid reduces glucose oxidation concomitantly with the increase in fatty acid oxidation. The most plausible explanation for this shift of substrate preference would be that referred to as the “Randle cycle” or “glucose-fatty acid cycle” [27]. To date, the Randle cycle has been interpreted to be largely due to the activation of pyruvate dehydrogenase kinase 4 (PDK4) and subsequent phosphorylation and inhibition of pyruvate dehydrogenase (PDH) by an elevated rate of FA oxidation [28], [29]. In this regard, the mechanism by which SCD1 overexpression restored the PA-induced decrease in glucose oxidation would be secondary to the decrease in FA oxidation. Because the PDK4 gene promoter contains a PPARα-response element (PRE) [30], activation of PPARα/PGC-1α complex by fatty acid ligand may play a role. This hypothesis is currently under investigation.

### Are the effects of SCD1 on cellular function cell-type specific?

In agreement with our data, the beneficial metabolic effects of SCD1 induction have been demonstrated in human primary arterial endothelial cells, skeletal muscle cells and pancreatic beta-cells [23], [31], [32]. These studies showed that SCD1 induction either by liver X receptor (LXR) activator TO-901317 or by palmitic acid contribute to improved insulin sensitivity, reduced endoplasmic reticulum (ER) stress and reduced inflammatory response while SCD1 induction is associated with increased intracellular lipid accumulation. In contrast, SCD1 deficiency has been shown to reduce ceramide synthesis in skeletal muscle and attenuates inflammation in white adipose tissue [25], [33].

In conclusion, this study shows that SCD1 in the heart is highly induced by an HS diet in vivo and by a variety of molecules relevant to metabolic syndrome in cardiac myocytes in vitro. Our results indicate that SCD1 induction clearly decreases palmitic acid-induced fatty acid oxidation, caspase 3 activation, apoptosis and mitochondrial ROS generation. These effects are associated with the accumulation of TG. These results presented here clearly argue against the concept that loss of SCD1 improves cardiac metabolism directly or indirectly through combating metabolic syndrome, and raise the intriguing possibility that SCD1 plays a distinct role in a cell type-dependent and a nutritional status-dependent manner.

## Materials and Methods

### Animals and treatments

Obesity and diabetes model rats were prepared as described previously [34]. Briefly, 6-week-old male Wistar rats (250–300 g) were divided into two groups of 6–8 rats: the control group was fed standard chow (MF: 81% carbohydrates, 13% protein, and 6% fat; Oriental Yeast Co., Tokyo, Japan), and the HS diet group was fed sucrose-rich chow (50% sucrose and 50% standard chow). All animals were housed according to institutional guidelines for 12 weeks in climate controlled metabolic cages with a 12-h light/12-h dark cycle, and food (as specified above) and water provided *ad libitum*. All experiments using these rats were approved by and performed according to the guidelines of the Animal Ethics Committee of Gunma University, Maebashi, Japan (*Permit Number*: 07-101).

### Hemodynamic measurements and echocardiographic evaluation

Ascending aortic pressure and heart rate were measured in the unrestricted, conscious state through a heparinized indwelling polyethylene catheter that was introduced into the left carotid artery 1 day before measurement. Data were analyzed using Power Lab and Chart v5.0.1 (AD Instruments, Castle Hill, Australia). Blood was collected after hemodynamic measurement and blood plasma was separated immediately by centrifuging at 1500 rpm for 20 min at 4°C. Left ventricular wall thickness and function were measured by echocardiography (EUB6000; Hitachi, Tokyo, Japan) using a 10-MHz probe. Before feeding and after 12 weeks of feeding, rats were anesthetized with ketamine (50 mg/kg) and xylazine (10 mg/kg) injected intraperitoneally and subjected to echocardiographic study. Left ventricular ejection fraction (EF, %), fractional shortening (FS, %) and the ratio of the early to late filling wave (E/A) of the transmitral pulse-wave Doppler velocity were measured as described previously.

### Tissue preparation and total RNA extraction

Following the induction of deep anesthesia, rats were killed and the hearts were excised immediately. Left ventricles were frozen rapidly in liquid nitrogen and stored at −70°C until real-time quantitative RT-PCR analysis. In addition, tissue blocks from hearts were fixed in 4% paraformaldehyde for 4 to 12 h, embedded in paraffin, and sectioned at 4-µm thickness for immunohistochemistry.

### RNA extraction and Real-Time Reverse Transcription (RT)-PCR

Total RNA was prepared from the heart using Isogen (Wako, Tokyo, Japan) according to the manufacturer's protocol. One microgram of RNA was used for reverse transcription with the RNA LA PCR Kit (TAKARA BIO, Shiga, Japan) and real-time RT-PCR analysis was performed using SYBR Green Real-time PCR Master Mix-Plus (TOYOBO, Osaka, Japan) according to the manufacturers' protocol. Primer sequences were as follows: rat SCD1-sense, 5′- CCTCATCATTGCCAACACCAT -3′; anti-sense, AGCCAACCCACGTGAGAGAA -3′. Specificity of the SYBR Green amplicons was confirmed by melting point analysis and gel electrophoresis in the presence of ethidium bromide (Nippon Gene, Tokyo, Japan). Expression of the housekeeping gene GAPDH was used for normalization.

### Immunohistochemistry

Human heart tissues were obtained from patients at autopsy with informed consent of their family at Gunma University Hospital. This protocol was approved by the Institutional Review Board at Gunma University Hospital. Tissue samples were fixed in 4% buffered formaldehyde and embedded in paraffin. Immunohistochemical staining was performed using a DAKO Catalyzed Signal Amplification (CSA) system (DAKO, Glostrup, Denmark), according to the manufacturer's instructions. Tissue cross sections (4 µm) including the left ventricle and interventricular septum were stained with rabbit monoclonal anti-SCD1 (C12H5) antibody (Cell Signaling Technology, MA, USA) at dilutions of 1∶200 in Can Get Signal immunostaining solution A overnight at 4°C. Counterstaining was performed with 2% methyl green. Negative controls were produced by substituting non-immune mouse immunoglobulin (DAKO) for the primary antibody.

### Measurements of plasma and heart tissue parameters

Plasma total cholesterol, HDL cholesterol, LDL cholesterol, TG, free fatty acid (FFA), glucose and insulin were determined by enzymatic, colorimetric assays by an external laboratory (SRL Inc., Tokyo, Japan). Tissue lipids were extracted using the method of Bligh and Dyer [35] and expressed as counts per minute in the whole organ. Fatty acid composition analysis in plasma and heart tissue was also performed by SRL Inc. The compositions were expressed as the concentration (µg/mL of plasma) and percentage by weight of total fatty acids. TG content in the heart was measured using TG-E Test Wako (Wako) according to the manufacturer's protocol.

### Preparation of neonatal rat cardiac myocytes

Primary neonatal rat cardiac myocyte cultures were prepared as previously described [36]. Using this method, we routinely obtained cardiac myocyte-rich cultures with >95% of the cells being cardiac myocytes, as assessed by immunocytochemical staining with monoclonal antibody against sarcomeric α-actinin (Sigma-Aldrich, MO, USA).

Serum-starved (24 h) cardiac myocytes were incubated in the presence or absence of glucose (100 to 450 mg/dl), insulin (1 to 10 µM), fatty acid-free BSA (Sigma-Aldrich)-conjugated palmitic acid, stearic acid, oleic acid and linoleic acid (each 100 µM, Sigma). Fatty acid composition adjusted for the HS diet group's parameters, was also stimulated (100 to 500 µM). After 24 h incubation at 37°C, mRNA was collected from the cells.

### Generation of Recombinant Adenovirus

Adenoviruses expressing LacZ (Ad-LacZ) or SCD1 (Ad-SCD1) were generated as described previously [37]. Cardiac myocytes were infected with Ad-SCD1 or Ad-LacZ at a multiplicity of infection of 20 for 48 h in preparation for the next experiment.

*Construction of siRNA oligonucleotide and transfection* -siRNA oligonucleotides (siSCD1: CCU UCU UGA GAU ACA CUC UGG, siGFP: GUU CAG CGU GUC CGG CGA GTT) were purchased from Hayashi Kasei (Osaka, Japan) and transient transfection of siRNA oligonucleotide was carried out using Lipofectamine RNAiMAX (Invitrogen Life Technologies, CA, USA) according to the manufacturer's protocol.

### Oil red O staining in cardiac myocytes

Neutral lipid within cardiac myocytes was detected by oil red O staining of fixed cells on gelatin-coated cover slips.

### Palmitic acid and glucose oxidation studies

Cellular palmitic acid or glucose oxidation rates were measured as described previously [38]. In brief, cardiac myocytes were prepared as above and then plated in approximately equivalent numbers in T25 flasks. Palmitic acid or vehicle control (Bovine serum albumin; BSA) exposure began at the switch to serum-free medium 48 h after Ad-SCD1 or Ad-LacZ infection. Seventeen hours later, the cells were treated with [^14^C]-palmitic acid or [^14^C]-glucose (Perkin Elmer, CT, USA), and #1 Whatman filter paper was suspended within each flask. The flasks were sealed, and 7 hours later the cells were lysed with 6N hydrochloric acid. The ^14^CO_2_ collected overnight on Whatman paper was liberated by alkalization with 2N sodium hydroxide, and was quantified by scintillation counting.

### Western blot analyses

Whole cell extracts were prepared using modified RIPA buffer containing 50 mM Tris-HCl (pH7.4), 150 mM NaCl, 1% Nonidet P-40, 0.25% sodium deoxycholate, 1 mM EDTA, and containing complete mini and phosSTOP solution (Roche, IN, USA). The mixture was rotated at 4°C for 15 min and centrifuged at 14000 rpm for 15 min. Western blot analysis was performed according to standard procedures using the following primary antibodies: rabbit monoclonal phosphor-AMPK, AMPK, phosphor-ACC, ACC, cleaved-caspase 3, caspase 3 mAb (Cell Signaling) and β-actin (Santa cruz biotechnology, CA, USA). Antigens were revealed by ECL (Amersham Biosciences, NJ, USA) after incubation with horseradish peroxidase-conjugated anti-mouse or anti-rabbit IgG.

### DAG and ceramide assay

Intracellular DAG and ceramide levels were measured using a DAG kinase assay as described previously with slight modification. Briefly, following 24 h incubation, lipids were extracted by modification of the method of Bligh and Dyer [35]. Samples were extracted with 3 ml chloroform/methanol (1∶2, v/v). Sufficient 1 M NaCl was added to bring the aqueous volume to 0.8 ml, and the monophase was mixed. Then, 1 ml CHCl_3_ and 1 ml of 1 M NaCl were added to the break phase and the phases were separated by brief centrifugation at 2000 rpm. The chloroform phase was analyzed within 72 h. Ceramide was converted to ceramide 1-[^32^P] phosphate by *E. coli* DAG kinase in the presence of [γ-^32^P] ATP. Labeled lipids (ceramide-1-phosphate) were separated by high performance thin-layer chromatography in chloroform∶methanol∶water (60∶35∶8, v/v). Following autoradiography, spots corresponding to ceramide-1-phosphate were scraped and radioactivity was counted using a scintillation counter. Quantification of DAG and ceramide was based on a standard curve of known amounts of DAG and ceramide. The changes in DAG and ceramide content were normalized based on total protein.

### Apoptosis assays

For detection of caspase-3 and caspase-7 activation, cardiac myocytes were plated in replicates of five in 96-well plates, transfected as described above and analyzed using the Caspase 3/7 Assay (Promega, WI, USA) according to the manufacturer's instructions. Samples were read after 8–24 h of incubation with the caspase substrate on a fluorescent plate reader.

For labeling nuclei of apoptotic cells, cardiac myocytes were plated on glass coverslips in 8-well slides, infected, fixed in 4% paraformaldehyde 48 h post-infection, and terminal deoxynucleotidyl transferase–mediated nick-end labeling (TUNEL) staining was performed using the *In Situ* Cell Death Detection Kit, Fluorescein (Roche) according to the manufacturer's protocol.

### Quantitative ROS detection

Intracellular ROS levels were detected with the oxidant-sensitive fluorogenic probes 5-(and-6)-chloromethyl-2′,7′-dichlorodihydrofluorescein diacetate, acetyl ester (CM-H_2_DCFDA, Invitrogen Life Technologies). Adenovirus infected or siRNA transfected cardiac myocytes on the 6-well dish were incubated with 10 µM CM-H_2_DCFDA in serum-free medium for 30 min at 37°C. Then, the cells were incubated in the presence or absence of palmitic acid or stearic acid for 20 min. After incubation at 37°C, fluorescence was detected.

### Effect of several humoral factors on SCD1 mRNA expression

Serum-starved (24 h) cardiac myocytes were incubated in the presence or absence of angiotensin II (0.1–1 µM), endothelin-1 (0.01–0.1 µM), norepinephrine (1–10 µM), lipopolysaccharide (10–50 ng/ml), hydrogen peroxide (0.1–50 µM) for 24 h, and hypoxic stress (3–9 h). After incubation at 37°C, mRNA was collected from the cells.

*Statistical analysis*—Values are reported as the mean ± SD. One way ANOVA was used to evaluate differences between groups. Where appropriate, post hoc multiple comparison tests were performed to evaluate differences between the control and experimental groups. A P value <0.05 was considered significant.
